# Human IgA-Expressing Bone Marrow Plasma Cells Characteristically Upregulate Programmed Cell Death Protein-1 Upon B Cell Receptor Stimulation

**DOI:** 10.3389/fimmu.2020.628923

**Published:** 2021-02-12

**Authors:** Annika Wiedemann, Marie Lettau, Ina Wirries, Annemarie Jungmann, Abdulrahman Salhab, Gilles Gasparoni, Henrik E. Mei, Carsten Perka, Jörn Walter, Andreas Radbruch, Andreia C. Lino, Thomas Dörner

**Affiliations:** ^1^ Department of Rheumatology and Clinical Immunology, Charité Universitätsmedizin Berlin, Berlin, Germany; ^2^ German Rheumatism Research Center Berlin (DRFZ), Berlin, Germany; ^3^ Department of Genetics and Epigenetics, Saarland University, Saarbrücken, Germany; ^4^ Department of Orthopedics, Charité Universitätsmedizin Berlin, Center for Musculoskeletal Surgery, Berlin, Germany

**Keywords:** plasma cells, B cell receptor, signaling, human bone marrow, B cells, CD19, IgA

## Abstract

The functions of bone marrow plasma cells (BMPC) beyond antibody production are not fully elucidated and distinct subsets of BMPC suggest potential different functions. Phenotypic differences were identified for human BMPC depending on CD19 expression. Since CD19 is a co-stimulatory molecule of the B-cell-receptor (BCR), and IgA^+^ and IgM^+^ BMPC express the BCR on their surface, we here studied whether CD19 expression affects cellular responses, such as BCR signaling and the expression of checkpoint molecules. We analyzed 132 BM samples from individuals undergoing routine total hip arthroplasty. We found that both CD19^+^ and CD19^−^ BMPC expressed BCR signaling molecules. Notably, the BCR-associated kinase spleen tyrosine kinase (SYK) including pSYK was higher expressed in CD19^+^ BMPC compared to CD19^−^ BMPC. BCR stimulation also resulted in increased kinase phosphorylation downstream of the BCR while expression of CD19 remained stable afterwards. Interestingly, the BCR response was restricted to IgA^+^ BMPC independently of CD19 expression. With regard to the expression of checkpoint molecules, CD19^−^ BMPC expressed higher levels of co-inhibitory molecule programmed cell death protein-1 (PD-1) than CD19^+^ BMPC. IgA^+^ BMPC characteristically upregulated PD-1 upon BCR stimulation in contrast to other PC subsets and inhibition of the kinase SYK abrogated PD-1 upregulation. In contrast, expression of PD-1 ligand, B and T lymphocyte attenuator (BTLA) and CD28 did not change upon BCR activation of IgA^+^ BMPC. Here, we identify a distinct characteristic of IgA^+^ BMPC that is independent of the phenotypic heterogeneity of the subsets according to their CD19 expression. The data suggest that IgA^+^ BMPC underlie different regulatory principles and/or exert distinct regulatory functions.

## Introduction

Human serum antibody titers are relatively stable and exhibit half-lives ranging from years to decades (1). They are produced by plasma cells (PC) located in the bone marrow (BM), which have a unique transcriptome with >70% of transcripts encoding the immunoglobulin heavy (IgH) and light (IgL) chains (2). Of these BMPC, a subset is considered to be long-lived producing IgG with half-live times above 10.000 years (1, 3).

We and others described a subset of BMPC lacking the costimulatory molecule CD19 (4–7). CD19 enhances signaling downstream of the B cell receptor (BCR) by lowering the threshold for its activation (8). Consistently, genetic loss of CD19 results in immune deficiency (9), while elevated CD19 expression has been detected in autoimmune diseases (10, 11). CD19^−^ BMPC display markers of advanced maturity and seem to contain a higher amount of long-lived PC than their CD19-expressing counterparts (4, 5), they persist after CD19-directed chimeric antigen receptor (CAR) T-cell therapy in B cell malignancies and produce IgG specific to tetanus toxoid, mumps and measles (12). In peripheral blood, low frequencies of CD19^low^/CD19^−^ plasmablasts have been identified during the acute response to influenza vaccination and steady state (6).

In contrast to previous assumptions that BMPC lack surface Ig (13), it was reported that IgA and IgM are present on the surface and functionally active (7, 14, 15). IgA^+^ and IgM^+^ BMPC have been shown to respond to BCR stimulation with spleen tyrosine kinase (SYK) and extracellular-signal regulated kinase (ERK) phosphorylation (14).

In this study, we show that BCR signaling is distinct among CD19^+^ versus CD19^−^ BMPC. Components of the BCR signaling pathway are expressed in both subsets, but the signaling kinase SYK and pSYK Y^352^ are higher expressed in CD19^+^ BMPC. Non-IgG BMPC react to BCR stimulation enhancing phosphorylation of BCR-associated kinases and characteristically upregulate the co-inhibitory molecule programmed cell death protein 1 (PD-1) in response to BCR ligation independently of their CD19 expression which can be abrogated using a SYK inhibitor. This indicates that IgA^+^ PC fulfill distinct immune functions likely related to antigen encounter and that their regulation involve PD-1 expression.

## Materials and Methods

### Donors

132 BM samples were obtained from patients (71 women, 61 men; 67 ± 10 years (mean ± SD); range, 41–92 years) undergoing total hip arthroplasty. The study was approved by the local ethics committee of Charité Universitätsmedizin Berlin in accordance with the declaration of Helsinki and written informed consent was obtained from all patients.

### Isolation of Mononuclear Cells

Mononuclear cells (MNCs) from the BM were isolated as described previously (4). Briefly, samples were fragmented, rinsed with PBS (Biochrom, Berlin, Germany) for cell culture and stimulation or with PBS/0.5%BSA/EDTA (PBE) (Miltenyi Biotech, Bergisch Gladbach, Germany) for staining, and filtered with a 70 µm cell strainer (BD Biosciences, Heidelberg, Germany). The obtained cell suspension was subjected to a density gradient centrifugation with Ficoll Paque (GE Healthcare, Buckinghamshire, UK) and/or used for whole BM stainings. The collected mononuclear cells were washed twice with PBS or PBE and either resuspended in RPMI 1640 (ThermoFisher Scientific, Waltham, MA, USA) for stimulation assays or in PBE for staining.

### Enrichment of Bone Marrow Plasma Cells by Magnetic-Activated Cell Sorting

MNCs from the BM were incubated with CD3-, CD14- and CD235a-microbeads and subjected to magnetic-activated cell sorting (MACS) according to the manufacturer’s protocol. Afterwards, cells were stained and sorted with a FACS Aria II (BD Biosciences). For some cell culture experiments, BMPC were enriched with CD138 microbeads according the manufacturer’s instructions.

### Whole Bone Marrow Staining

Filtered cell suspension was lysed with prewarmed 1× Lyse/Fix buffer (BD Biosciences). After centrifugation and washing with ice-cold PBS, cells were permeabilized with Perm Buffer II (BD Biosciences) according to the manufacturer’s protocol and as described previously (16). Subsequently, the cells were washed with PBE and stained intracellularly.

### Stainings for Flow Cytometry

2 × 10^6^ MNCs were surface-stained for 15 min at 4°C or for 1 h at room temperature (RT) for intracellular staining with different combinations of antibodies (**Table S1**). DAPI (Molecular Probes, Eugene, USA) or LIVE/DEAD Fixable Blue Dead Cell Stain Kit (ThermoFisher Scientific) were used to exclude dead cells according to the manufacturer’s protocol. Cells were analyzed with a FACS Canto II or LSR Fortessa X-20 flow cytometer in standard configuration (BD Biosciences). PC, BC and T cells were identified as previously described (17) (**Figure S1**) and BMPC subsets were sorted. For quality control, Cytometer Setup & Tracking Beads (BD Biosciences) and SHPERO Calibration Particles (BD Biosciences) were used to get reproducible median fluorescence intensities (MFI). Staining controls were either done by fluorescence minus one (FMO) or isotype control approach.

For assessment of absolute counts, cells were resuspended in a defined volume after staining and 20 µl of CountBright^™^ Absolute Counting Beads (ThermoFisher Scientific) were added before acquisition. Absolute cell counts were calculated as follows:

$$\frac{\text{recorded cell events}}{\text{recorded bead events}}\;\times \frac{\text{beads in }20\text{ µl}}{\text{volume of cell suspension}}\;=\;\text{cells}/\text{µl}$$

### Microarray for Transcriptome Analysis

Analysis of transcriptomes of CD19^+^ and CD19^−^ BMPC was performed as previously described (4) (GEO accession number GSE56464).

### RNA Library Preparation

RNA of CD19^+^IgA^+^, CD19^+^IgA^−^, CD19^−^IgA^+^, and CD19^−^IgA^−^ sorted cells was isolated by using the RNeasy Micro Kit (Qiagen, Hilden, Germany) following the manufacturer’s instructions.

A modified SmartSeq 2 protocol was performed; total RNA between 2 and 10 ng was used as input for the reverse transcription. RNA was primed by adding 1 µM Oligo-dT Primer (final conc.; 5′AAGCAGTGGTATCAACGCAGAGTACTTTTTTTTTTTTTTTTTTTTTTTTTTTTTTVN; V = A/C/G, N = any base), 1 mM dNTPs (final conc.) followed by a denaturation at 72°C for 3 min and immediately cooling on ice. Reverse transcription was performed in a 10-µl volume reaction by using 0.5 µl Superscript II RT (200 U/µl, Thermo Fisher Scientific), 0.4 µl RNAse inhibitor (40 U/µl, Promega, Madison, USA), 5 mM DTT, 1 M Betaine, 6 mM MgCl2 and 1 µM TSO (B-AAGCAGTGGTATCAACGCAGAGTACAT997, B = 5′ biotin, 7 = LNA g, 9 = RNA-G) under the following incubation conditions: 42°C for 90 min, 10× cycling of 50°C for 2 min and 42°C for 2 min, finalized by 70°C for 15 min. The preamplification of the cDNA was carried out by utilizing the KAPA HiFi HotStar Ready Mix (Roche, Basel, Switzerland) and 0.1 µM of the IS PCR primers (5´AAGCAGTGGTATCAACGCAGAGT) in a 25 µl volume reaction under the following PCR conditions: 98°C for 3 min, 10× cycling of 98°C for 20 s, 67°C for 15 s, 72°C for 6 min and a final elongation at 72°C for 5 min. The cDNA was purified by the use of 1× Agencourt AMPure XP Beads (Beckman Coulter, Brea, CA, USA), quantified by the help of Qubit^®^ dsDNA HS Assay Kit (Thermo Fisher Scientific) and the cDNA integrity was examined *via* the analysis of the fragment size distribution by using the Agilent 2100 Bioanalyzer (High Sensitivity DNA Analysis Kit, Agilent, Santa Clara, CA, USA).

The libraries were prepared by applying a tagmentation based approach using the Nextera DNA Library Preparation Kit (Illumina, San Diego, CA, USA). 8 ng of each cDNA were tagmented for 10 min at 55°C by the use of 1 µl of the Tagment DNA Enzyme 1 in a 20 µl reaction pursued immediately by the purification of the tagmented fragments by the use of the MinElute PCR Purification Kit (Qiagen) following the manufacturer’s instructions. The amplification of the libraries was performed in a 30 µl reaction using the NEBNext High-Fidelity 2× PCR Master Mix (New England Biolabs, Ipswich, MA, USA) and 0.33 µM indexed adapters (5′AATGATACGGCGACCACCGAGATCTACAC[i5]TCGTCGGCAGCGTC and 5′CAAGCAGAAGACGGCATACGAGAT[i7]GTCTCGTGGGCTCGG; Illumina). 8 PCR cycles were done of the following PCR program: 75°C 5 min, 98°C 10 s, cycling of 98°C 30 s, 63°C 30 s and 7°C 1 min, finalized by a long elongation at 72°C for 7 min. The libraries were purified by utilizing 0.9× Agencourt AMPure XP Beads (Beckman Coulter), the DNA concentrations were quantified by the help of the Qubit^®^ dsDNA HS Assay Kit (Thermo Fisher Scientific) and the size distribution of the amplified fragments was examined by the use of the Agilent 2100 Bioanalyzer (High Sensitivity DNA Analysis Kit, Agilent). The libraries were sequenced on the Illumina HiSeq 2500 by using the 100 bp single read sequencing mode.

### mRNA Seq Data Processing

Adapter-sequences of FastQ format RNA-seq reads were removed and the reads were trimmed of low quality ends (phred score = 20) by the use of Trim Galore! (version 0.4.2) (“Babraham Bioinformatics - Trim Galore!” 2017). The reads were aligned to the hg38 reference genome (Genbank: GCA_000001405.15) by using grape-nf (version 433e7621f6) (18), which combines STAR (version 2.4.0j) (19) for the alignment and RSEM (version 1.2.21) (20) for the read assignment.

### B Cell Receptor Stimulation

For short-term kinetics, 2 × 10^6^ MNCs were equilibrated with RPMI 1640 at 37°C for 30 min and stimulated with 30 µg/ml anti-IgM/IgG/IgA (Jackson ImmunoResearch, Ely, UK), 10 µg/ml anti-IgA (Jackson ImmunoResearch) or 10 µg/ml anti-IgG (Jackson ImmunoResearch) for the indicated times. To assess baseline phosphorylation (0 min), cells were treated with RPMI for 5 min. Reaction was stopped using 1× Lyse/Fix Buffer (BD Biosciences) and cells were permeabilized with Perm Buffer II (BD Biosciences) according to the manufacturer’s protocol, washed with PBE and stained intracellularly. In some experiments, cells were pre-incubated with SYK inhibitor entospletinib (GS-9973, Selleck Chemicals, final concentration 10 µM), BTK inhibitor acalabrutinib (ACP-196, Selleck Chemicals, final concentration 100 µM) or DMSO as control for 1h prior to stimulation with anti-BCR.

For cell culture, 2 to 3 × 10^6^ MNCs were seeded in a 96-Well plate (BD Falcon) with medium as described in the final incubation step for long-lived PC (21) including interleukin (IL-) 6 and a proliferation-inducing ligand (APRIL). 1 µg/ml anti-BCR F(ab)_2_ IgM/IgG/IgA (Jackson ImmunoResearch) or 0.5 µg/ml crosslinked CD40L (Miltenyi) were added to the culture and cells were cultured at 37°C and 5% CO_2_ in a humidified incubator for up to four days. In some experiments, cells were pre-incubated with SYK inhibitor entospletinib (final concentration 10 µM) or DMSO as control for 30 min prior to stimulation with anti-BCR. Concentrations of stimulating agents and inhibitors were titrated in previous experiments.

### Data and Statistical Analysis

Flow cytometric data was analyzed with FlowJo version 7.6.5 or 10.1 (FlowJo LLC, BD Biosciences). Statistical analysis was performed with GraphPad Prism 6 or 8 software (Graphpad Software, San Diego, CA, USA). Statistical significance was tested as indicated.

## Results

### CD19+ and CD19− Bone Marrow Plasma Cells Express B Cell Receptor-Associated Molecules

**Figure 1:**
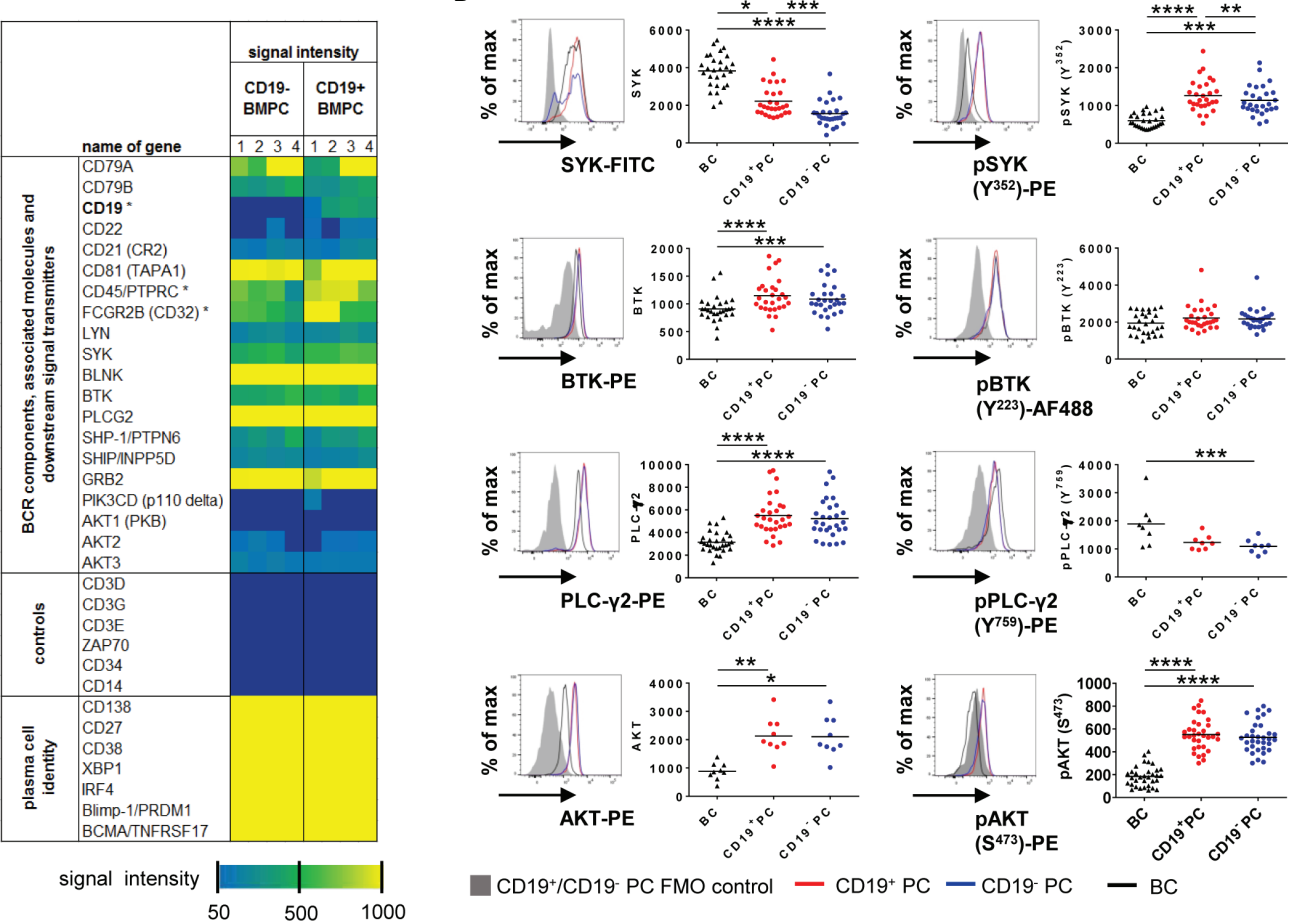
CD19^+^ and CD19^−^ BMPC express BCR-associated kinases. **(A)** Gene expression profiling of CD19^+^ and CD19^−^ BMPC. Expression of BCR components and molecules involved in intracellular BCR signaling in CD19^+^ and CD19^−^ BMPC. Dark blue indicates absent calls. Asterisks indicate significantly different expression (Fold change CD19^−^ vs CD19^+^ for CD19: −11, 87; CD45: −1, 67; CD32: −1, 46). **(B)** Median fluorescence intensities of the kinases SYK, BTK, PLCγ2 and AKT and their baseline phosphorylation among CD19^+^, CD19^−^ BMPC and CD27^+^CD38^−^ BMBC. Representative histogram of CD19^+^ (red lines) and CD19^−^ (blue lines) BMPC as well as CD27^+^CD38^−^ BMBC staining (black lines) together with a FMO control (filled grey histogram). Bar represents mean, ****p < 0.0001, ***p < 0.001, **p < 0.01, *p < 0.05 by Friedman-Test with Dunn’s correction for multiple comparisons. SYK/pSYK (Y^352^): n = 28; PLC-γ2: n = 29, pPLC-γ2 (Y^759^): n = 8, BTK/pBTK (Y^223^): n = 28, AKT: n = 9, pAKT (S^473^): n = 33.

To assess whether CD19^+^ and CD19^−^ BMPC both have the prerequisites for BCR signaling, CD19^+^ and CD19^−^ BMPC gene expression was analyzed for BCR-associated molecules (GEO accession number GSE56464) (4). Plasma cell phenotype as well as presence or absence of the CD19 transcript could be confirmed and classical markers of other lineages were not detected (**Figure 1A**). Regarding the capability of BCR signaling, the analysis showed that transcripts of molecules relevant for BCR signaling are found in both CD19^+^ and CD19^−^ BMPC, among them e.g. BCR components (CD79A, CD79B), BCR co-stimulating receptors (CD21, CD81), protein tyrosine kinases (PTKs) and protein serine/threonine kinases (PSKs, SYK, PLCG2, BTK, AKT3) but also BCR associated phosphatases (PTPN6, INPP5D). In this analysis, a similar expression of the aforementioned molecules was found for both CD19^+^ and CD19^−^ PC on the mRNA level.

Next, we measured protein expression of representative downstream PTKs (SYK, BTK and PLC-γ2) as well as AKT1/2/3 (panAKT) by flow cytometry and compared their baseline (tonic) phosphorylation in CD19^+^ and CD19^−^ BMPC with CD27^+^CD38^−^ BMBC as a control (**Figure 1B**). Cells were gated according to the strategy showed in **Figure S1**. Significant differences were detected for the expression of SYK and pSYK Y^352^ in contrast to the transcriptome data: CD19^−^ BMPC showed a significantly lower median fluorescence intensities (MFI) of SYK and pSYK, which is downstream of LYN as one of the proximal BCR kinases. For the PTKs BTK and PLC-γ2 further downstream of SYK in the BCR signaling pathway and for the PSK AKT, which plays a role in survival downstream of PI3K, we found a similar expression between the two BMPC subsets. Likewise, there was no difference between the two PC subsets for pBTK Y^223^, pPLC-γ2 Y^759^, and pAKT S^473^. Overall, CD27^+^CD38^−^ BMBC displayed a lower expression of PTKs except for SYK protein expression and pPLC-γ2 Y^759^. These findings emphasize that SYK and pSYK may play a distinct functional role in different BMPC subsets.

### B Cell Receptor Stimulation Induces Phosphorylation of B Cell Receptor-Associated Kinases in Bone Marrow Plasma Cell Subsets

**Figure 2:**
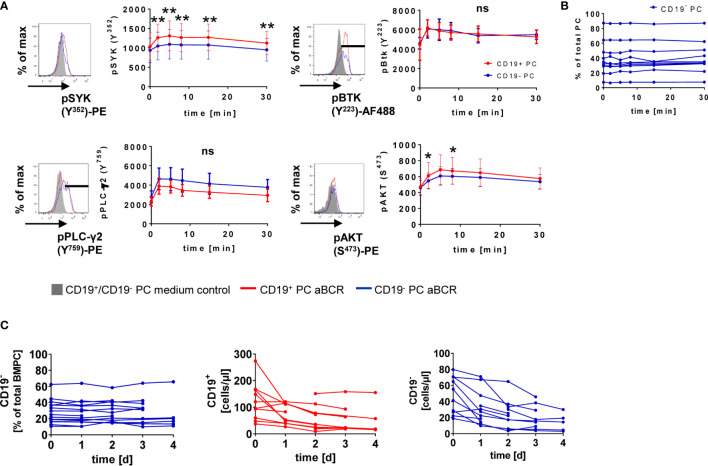
CD19^+^ and CD19^−^ BMPC respond to BCR stimulation. **(A)** MNCs isolated from the BM were stimulated with 30 µg/ml anti-BCR F(ab)_2_ IgM/IgG/IgA (aBCR) for the indicated times and stained intracellularly for the respective phosphosites of different PTKs or PSK. A representative histogram is shown for each pPTK/pPSK (5′ RPMI/5′ aBCR). For pSYK and pAKT, the median fluorescence intensity of total CD19^+^ or CD19^−^ BMPCs is displayed. High expressors were gated for pBTK and pPLC-γ2 and the MFI is shown for this population. **p < 0.01, *p < 0.05 by Wilcoxon signed rank test. ns, not significant. pSYK: n = 10 (t = 15 min, n = 9); pBTK: n = 10; pPLC-γ2: n = 4; pAKT: n = 10. Data are presented as mean ± SD. **(B)** Frequency of CD19^−^ PC among total BMPC during short-term BCR kinetics. **(C)** BM MNCs were cultured for up to four days in the presence of 1 µg/ml anti-BCR F(ab)_2_ IgM/IgG/IgA (aBCR) or medium (control). Frequency of CD19^−^ BMPC among total PC (n = 6–16) and absolute counts of CD19^+^ and CD19^−^ BMPC (n = 5–12) during the culture with anti-BCR.

IgA^+^ and IgM^+^ BMPC react to BCR stimulation (14) but whether CD19 expression is related to differential responsiveness remained unclear. We found that phosphorylation of SYK, BTK, PLC-γ2 and AKT is induced after BCR stimulation (**Figure 2A**). For CD19^+^ and CD19^−^ PC, phosphorylation reached its maximum after 2 min (pBTK Y^223^, pPLC-γ2 Y^759^) or 5 min (pSYK Y^352^, pAKT S^473^) (**Figure 2A**). Significant differences between CD19^+^ and CD19^−^ BMPC during BCR stimulation kinetics were found for all time points after stimulation when looking at SYK phosphorylation with consistently higher values for CD19^+^ BMPC and pAKT at early time points (2 and 8 min). The BCR response of CD27^+^CD38^−^ BMBC (16, 22) served as positive and T cells as negative control (**Figure S2**).

We wondered if BCR stimulation might lead to a loss of CD19 expression possibly related to a differentiation of CD19^+^ BMPC into CD19^−^ PC. During the short-term stimulation of 30 min, no change of the frequency of CD19^−^ BMPC could be detected (**Figure 2B**). More importantly, we observed no changes in the frequency of CD19^−^ BMPC during a four-day culture upon anti-BCR stimulation (**Figure 2C**). Absolute numbers of CD19^+^ and CD19^−^ BMPC decreased during the first day of culture but remained stable afterwards (**Figure 2C**) indicating that the relative proportion and the expression of CD19 did not change.

### IgA^+^ but Not IgG^+^ Bone Marrow Plasma Cells Respond to B Cell Receptor Stimulation

Since surface BCR expression on total BMPC has been described before (7, 14, 15), we addressed how the differences between CD19^+^ and CD19^−^ BMPC and their distinct BCR reactivity might be related to their isotype expression.

**Figure 3:**
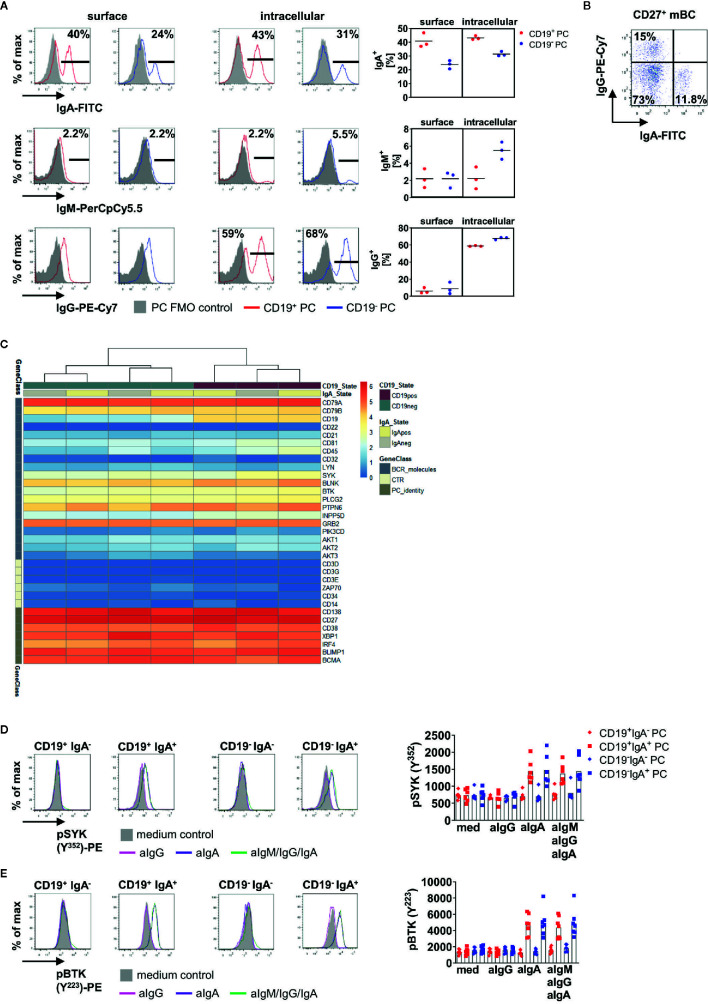
IgA^+^ BMPC are responsible for enhanced phosphorylation of PTKs after BCR stimulation. **(A)** Surface and intracellular staining of IgA, IgM and IgG in BMPC. Representative histograms of one out of three experiments (left), bar indicates gate for Ig^+^ cells. Filled grey histograms indicate the PC FMO control, the solid line indicates staining of Ig. Numbers indicate mean of the frequency of Ig^+^ among each subset (n = 3). Frequencies for surface and intracellular Ig expression from three experiments (right), bar indicates mean. **(B)** IgA and IgG surface staining of CD27^+^CD38^−^ memory BMBC. **(C)** mRNA expression for BCR associated genes in CD19^+^IgA^+^, CD19^+^IgA^−^, CD19^−^IgA^+^ and CD19^−^IgA^−^ BMPC shown by log_2_(CPM+1) values. n = 2. **(D, E)** BM MNCs were either stimulated with 10 µg/ml anti-IgA F(ab)_2_ (aIgA), 10 µg/ml anti-IgG F(ab)_2_ (aIgG) or 30 µg/ml anti-BCR F(ab)_2_ IgM/IgG/IgA (aIgM/IgG/IgA) for 5 min and stained for pSYK (Y^352^) (**D,** n = 5–6) or pBTK (Y^223^) (**E,** n = 7). A representative histogram is shown for each subset (solid lines: 5′anti-BCR; filled grey histogram: 5′RPMI). Significance was tested with Friedman-Test with Dunn’s correction for multiple comparisons.

First, we compared surface and intracellular expression of IgA, IgM and IgG on CD19^+^ and CD19^−^ PC (**Figure 3A**). IgA^+^ PC were found more frequently among CD19^+^ BMPC whereas more than half of CD19^−^ BMPC expressed IgG. We could also detect comparable frequencies of IgA^+^ cells with surface membrane and intracellular staining methods (surface: CD19^+^ 40.1%, CD19^−^ 23.9%, intracellular: CD19^+^ 43.1%, CD19^−^ 31.4%), whereas for IgG, no surface expression could be detected. Intracellular staining of IgG revealed that 58.8% of CD19^+^ PC expressed IgG and 67.8% of CD19^−^ PC. As control, IgG surface expression was readily detectable on CD27^+^ BMBC in the same staining (**Figure 3B**). IgM was only detectable at very low frequencies on human BMPC subsets (~2% of CD19^+^/CD19^−^ PC). Thus, BCR surface expression was largely limited to IgA^+^ PC and a substantially lower IgM^+^ fraction.

Next, we addressed if BMPC defined by CD19 and IgA expression display a unique expression of BCR related molecules. To do so, we sorted CD19^+^IgA^+^, CD19^+^IgA^−^, CD19^−^IgA^+^ and CD19^−^IgA^−^ and analyzed their transcriptomes using RNA Seq. Purity of the sorted populations was checked after the sort by flow cytometry (**Figure S3A**) as well as by CD19 and Ig isotype expression in the sorted samples (**Figure S3B**) validating the sorting parameters. PC identity could be confirmed by expression of e.g. CD138, BLIMP1, and IRF-4 (**Figure 3C**) and the J-chain was expressed in all four subsets. We analyzed BCR associated molecules in the four subsets after mRNA sequencing (**Figure 3C**) and found that all four subsets defined by IgA and CD19 express BCR associated molecules on the transcriptional level.

To address if BCR reactivity was linked to isotype expression in CD19^+^ and CD19^−^ BMPC, we stained pSYK Y^352^ or pBTK Y^233^ together with IgA after BCR stimulation. Of interest, these experiments revealed that IgA^+^ PC are responsible for increased phosphorylation in both CD19^+^ and CD19^−^ PC (**Figures 3D, E**). After stimulation with anti-IgM/IgG/IgA, the MFIs of pSYK and pBTK increased in CD19^+^IgA^+^ and CD19^−^IgA^+^ PC, whereas CD19^+^IgA^−^ and CD19^−^IgA^−^ PC did not show increased MFIs of pPTKs. This result was confirmed by stimulation with anti-IgG or anti-IgA alone. Stimulation with anti-IgA led to similar MFIs of pSYK Y^352^ and pBTK Y^223^ in both CD19^+^IgA^+^ and CD19^−^IgA^+^ PC compared to stimulation with anti-IgM/IgG/IgA, whereas for stimulation with anti-IgG alone, no increase of pPTK MFI was detected. When comparing the response of CD19^+^IgA^+^ and CD19^−^IgA^+^ PC, no significant difference was observed.

Pre-incubation with SYK and BTK inhibitors and subsequent anti-IgA stimulation resulted in reduced phosphorylation of pSYK and pBTK in both CD19^+^IgA^+^ and CD19^−^IgA^+^ PC compared to control. The SYK inhibitor entospletinib led to a significant reduction of 68% (CD19^+^IgA^+^) and 62% (CD19^−^IgA^+^) (**Figure S3C** **, left**), respectively. The BTK inhibitor acalabrutinib resulted into a significant reduction of 35% (CD19^+^IgA^+^) and 40% (CD19^−^IgA^+^) (**Figure S3C** **, right**), respectively suggesting a prominent role for SYK in BMPC BCR signaling.

We further compared the stimulation response of the IgA1- and IgA2-expressing subpopulations. No difference between IgA1 and IgA2-expressing cells inside each CD19 subset was found in terms of pSYK Y^352^ and pBTK Y^223^ phosphorylation, respectively or for IgA1- or IgA2-expressing cells between CD19^+^ and CD19^−^ PC (**Figure S3D, E**). Of note, the majority of IgA-expressing cells among BMPC were of the IgA1 isotype (CD19^+^ 46 ± 10%, CD19^−^ 30 ± 11% of total PC) and as little as 4% of total PC expressed IgA2 in each subset (**Figure S3F**).

### Inhibitory Molecules Are Expressed by Bone Marrow Plasma Cell Subsets

**Figure 4:**
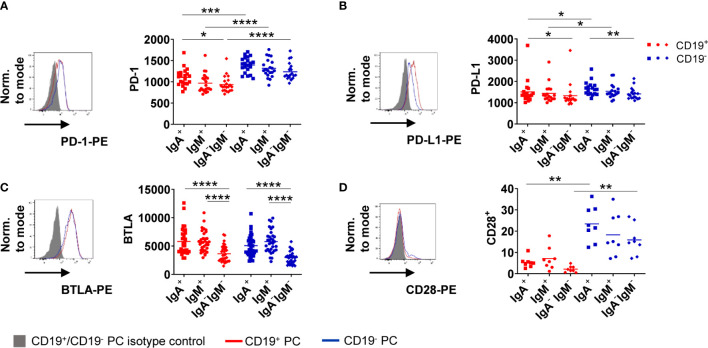
CD19^−^ BMPC express higher levels of PD-1 than CD19^+^. Baseline expression of PD-1 (**A**, MFI), PD-L1 (**B**, MFI), BTLA (**C**, MFI) and CD28 (**D**, frequency) was assessed in IgA^+^, IgM^+^ and IgA^−^IgM^−^ BMPC. A representative histogram with the respective isotype control staining (filled grey histogram: isotype control, solid lines: staining of CD19^+/−^ BMPC) and cumulative data is shown (n = 8–33) for IgA^+^, IgM^+^ and IgA^−^IgM^−^ BMPC. Bars indicate mean. ****p < 0.0001, ***p < 0.001, **p < 0.01, *p < 0.05 by Friedman-Test with Dunn’s correction for multiple comparisons.

Activation of B cells *via* the BCR leads to upregulation of activating as well as inhibitory markers to counterbalance immune activation, like e.g. checkpoint molecules (23). We asked if these molecules are functionally related to BCR stimulation in BMPC as B cells in the periphery upregulate PD-1 upon *in vitro* stimulation (24). First, we assessed expression of PD-1, PD-1 ligand (PD-L1), B and T lymphocyte attenuator (BTLA), T cell immunoglobulin and mucin-domain containing-3 (TIM-3) and Lymphocyte-activation gene 3 (LAG-3) as well as CD28 at baseline (**Figure 4**). We observed higher expression of PD-1 on CD19^−^ BMPC compared to CD19^+^ BMPC independently of their isotype (**Figure 4A**). PD-L1, a ligand of PD-1, was also found to be somewhat higher expressed on CD19^−^ compared to CD19^+^ PC. PC without Ig surface expression expressed lower levels than PC subsets with Ig surface expression (**Figure 4B**). Moreover, BMPC subsets expressing IgA or IgM on their surface showed significantly higher BTLA expression compared to BMPC lacking Ig surface expression at baseline independently of their CD19 expression (**Figure 4C**). We did not detect any TIM-3 or LAG-3 expression in human BMPC (data not shown). The co-stimulatory molecule CD28 was found to be higher expressed in CD19^−^ BMPC of all isotypes (**Figure 4D**). The distinct baseline expression of PD-1, PD-L1, BTLA, and CD28 indicate that certain BMPC subsets follow different functional directions.

### IgA^+^ Bone Marrow Plasma Cells Uniquely Upregulate Programmed Cell Death Protein-1 Following B Cell Receptor Stimulation

**Figure 5:**
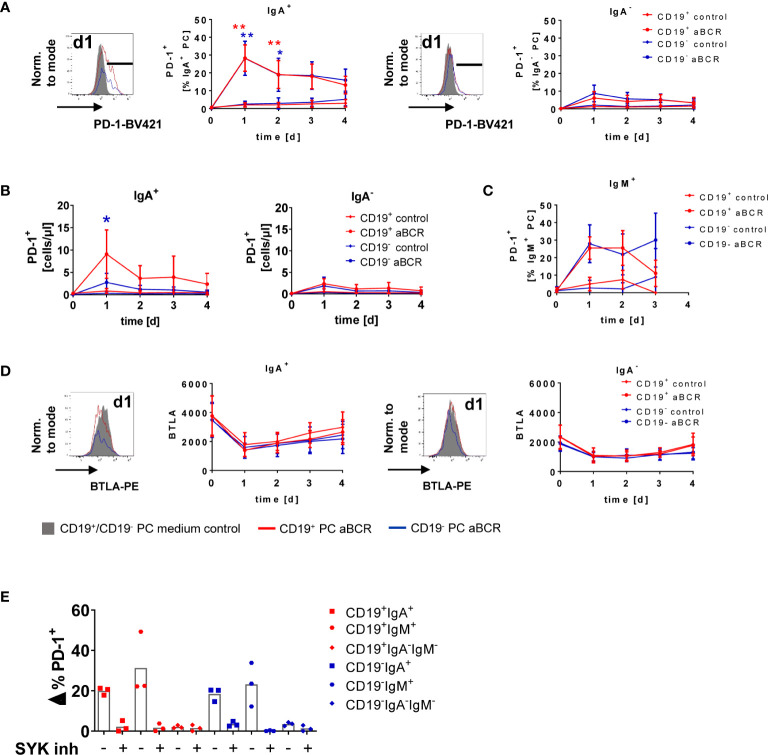
PD-1 is upregulated on IgA-expressing BMPC following BCR stimulation. BM MNCs were cultured for up to four days in the presence of 1 µg/ml anti-BCR F(ab)_2_ IgM/IgG/IgA (aBCR) or medium (control). **(A)** A representative histogram of PD-1 is shown (filled grey: staining d1 medium control; solid lines: staining d1 aBCR). PD-1 expression on IgA^+^ (left) and IgA^−^ (right) BMPC was analyzed among the subsets defined by CD19. Mean ± SD; n = 6–16 for each timepoint, Kruskal-Wallis-Test with Dunn’s correction for multiple comparisons. *p < 0.001, **p < 0.0001. **(B)** Absolute cell counts of PD-1^+^ cells during the culture with anti-BCR among IgA^+^ (left) and IgA^−^ (right) BMPC of CD19^+^ and CD19^−^ BMPC. Mean ± SD; n = 5–12 for each timepoint, Kruskal-Wallis-Test with Dunn’s correction for multiple comparisons. *p < 0.001. **(C)** PD-1 expression on IgM^+^ BMPC was analyzed among the subsets defined by CD19. Mean ± SD; n = 4–9 for each timepoint, Kruskal-Wallis-Test with Dunn’s correction for multiple comparisons. **(D)** Representative histogram (d1 medium control and aBCR) of BTLA (MFI) and cumulative data of IgA^+^ and IgA^−^ CD19^+/−^ BMPC after BCR stimulation. n = 2–16 for each timepoint. **(E)** BM MNCs were incubated with SYK inhibitor entospletinib or DMSO as a control 30 min prior to stimulation with anti-BCR for 1 day and stained for PD-1 together with CD19, IgA and IgM. Frequency of PD-1^+^ cells from stimulated samples minus unstimulated controls (Δ%) for the BMPC subsets defined by CD19, IgA and IgM, bar indicates mean, n = 3.

To address the functional outcome of BCR stimulation of BMPCs, we cultured BM MNCs for up to four days with anti-BCR or CD40L as control (**Figure 5**, **Figure S4**). Here, PD-1 was upregulated by 28.3 ± 7.4% (CD19^+^) and 28.2 ± 9.6% (CD19^−^) of IgA-expressing BMPC following BCR stimulation compared to medium control (**Figure 5A** **, left**) without signs of proliferation (data not shown). This upregulation was maximal on day 1, declined towards day 2 and remained stable until the end of the study (day 4), but was still higher than in the medium control. BMPC lacking IgA on the surface did only show upregulation of PD-1 by less than 10% (**Figure 5A** **, right**). Assessment of the absolute number of PD-1^+^ cells after BCR stimulation revealed a similar pattern as seen for the frequency: IgA-expressing cells showed enhanced numbers of PD-1–expressing cells, whereas in IgA^−^ BMPC, only a minor increase was detected (**Figure 5B**). Of note, the few IgM^+^ BMPC analyzed also upregulated PD-1 due to BCR stimulation (**Figure 5C**). After incubation with CD40L alone as a control, PC did not upregulate PD-1 (<5%) (**Figure S4A**) suggesting that this upregulation is restricted to BCR stimulation. Combined stimulation of the BCR and CD40 did not lead to increased PD-1 frequencies compared to stimulation of the BCR alone (data not shown). Staining for the checkpoint molecules BTLA and PD-L1 after BCR stimulation showed no upregulation of the respective molecules on BMPC but BTLA expression decreased independently of the culture condition (**Figure 5D**, **S4B**). CD28 expression, which was found to be enhanced on CD19^−^ BMPC at baseline, did not change upon BCR stimulation (**Figure S4C**). When seeding BMPC enriched by positive selection with CD138 microbeads under the same incubation conditions, we observed upregulation of PD-1 upon BCR stimulation on IgA^+^ cells in both CD19^+^ and CD19^−^ PC (**Figure S4D**). In this experiment, 74.5% of the cell suspension represented PC, whereas prior to enrichment, the percentage was as low as 1.34% (**Figure S4D**). Taken together, IgA-expressing BMPC are not only capable of signaling *via* BCR-associated kinases, but also able to upregulate the co-inhibitory molecule PD-1, a characteristic function in response to BCR stimulation.

### Inhibition of Spleen Tyrosine Kinase Abrogates Programmed Cell Death Protein-1 Upregulation in Human Bone Marrow Plasma Cell

SYK and pSYK were significantly higher expressed in CD19^+^ compared to CD19^−^ BMPC, and the SYK inhibitor entospletinib could substantially diminish phosphorylation of SYK due to BCR stimulation. Since we observed upregulation of PD-1 on IgA-expressing PC after one day of BCR stimulation in human BMPC cultures, we wondered if SYK might interfere with this effect. For this purpose, we incubated BM MNCs with entospletinib prior to BCR stimulation. Indeed, we could detect a major reduction of PD-1^+^ cells among IgA^+^ and IgM^+^ BMPC due to the SYK inhibitor which was comparable to the frequencies in the medium control (**Figure 5E**). This is reflected in 96.75% (CD19^+^IgA^+^), 86.5% (CD19^−^IgA^+^), 92.4% (CD19^+^IgM^+^) and 99.13% (CD19^−^IgM^+^) of inhibition. For IgA^−^IgM^−^ BMPC, comparable values were found for the different incubation conditions. Thus, SYK still plays a major role in BCR signal transduction in human BMPC.

## Discussion

The function of BMPC in addition to antibody production is not yet fully understood. It is commonly believed that the BCR is not expressed on BMPC and that no remaining function is associated with its expression.

Here we analyzed the downstream BCR signaling in human BMPC together with CD19 expression. We found that BCR responsiveness of BMPC did not uniquely depend on CD19 expression as a major costimulatory molecule of BCR signaling (25) as BCR-associated molecules were found on the mRNA level for both CD19^−^ and CD19^+^ subsets (**Figures 1** and **3**). Notably, CD19 expression was not influenced by BCR stimulation and remained stable *in vitro* (**Figure 2C**). This result suggests that BCR engagement does not impact on CD19 expression on BMPC. Until now, to our knowledge, no mechanism that leads to a distinct expression of CD19 on BMPC has been proposed, which allows the hypothesis that both subsets are generated simultaneously.

SYK and pSYK Y^352^ were higher expressed in CD19^+^ BMPC at baseline and after stimulation. During BCR stimulation, SYK has been reported to play a key role in transmission of the activation signal into the cell (26, 27). This indicates that SYK in contrast to BTK appears to be also crucial for transmitting signals in PCs.

When taking Ig isotype expression into account, IgA^+^ BMPC in both CD19^+^ and CD19^−^ BMPC showed enhanced protein kinase phosphorylation after BCR activation which confirms prior findings that IgA-(and IgM)-expressing BMPC are able to react to BCR stimulation (14). Since CD19^+^ BMPC contain more IgA-expressing cells than their CD19^−^ counterparts, it provides the explanation of higher phosphorylation in response to BCR stimulation when only CD19 expression during stimulation kinetics has been considered.

In contrast to our initial hypothesis that CD19 modulates BCR responsiveness in PC, similar phosphorylation of PTKs by IgA^+^ BMPC was detected independently of CD19 expression (**Figures 3D, E**). Similarly, in mice, the presence or absence of CD19 did not have an impact on the calcium response induced by IgM-crosslinking (25). In another study, two functionally distinct populations of murine BMPC defined by the Ig isotype (IgM and IgG) were described: IgM^+^ BMPC displayed a modified gene expression profile and enhanced production of CCL5 and IL-10 after antigenic boost, whereas IgG^+^ BMPC did not express a functional BCR (15). However, these results might not be directly translatable to human BM since only around 2% to 5% of human BMPC express IgM. In human BMPC, BCR crosslinking led to increased phosphorylation of SYK and ERK in IgA^+^ and IgM^+^ BMPC as well as to enhanced calcium influx and survival *in vitro* (14). When we included Ig isotypes in our analysis of BCR responsiveness, we saw that IgA^+^ (and the few IgM^+^) BMPC responded to BCR stimulation independently of the presence of absence of CD19.

IgA-expressing cells comprise 30% to 65% in the CD19^+^ and around 15% to 56% in the CD19^−^ BMPC subset in our cohort. It was described that rotavirus-specific IgA^+^ BMPC were present decades after exposure of individuals to the virus and thus can most likely also be considered as long-lived (7). In humans, both CD19^+^ and CD19^−^ PC are found in the gut and the majority of them express IgA in both subsets (28). Monomeric IgA is generated due to activation of B cells in the lymph nodes, whereas polymeric IgA is induced in the gastro-intestinal tract. In the transcriptomes of the BMPC analyzed, we found the J-chain to be expressed, which thus would favor induction in the gut. A less stringent compartmentalization between systemic and mucosal immunity has been suggested after discovering that murine IgA^+^ PC generated by oral immunization in the gut can be found for up to three months in the BM (29). In mice, certain commensal bacteria are able to induce enhanced levels of serum IgA as well as the presence of IgA-expressing PC in the BM (30). However, current evidence does not exclude that IgA versus IgG BMPC are generated during the same immune activation but taking different routes of B cell activation using possibly distinct checkpoint molecule and cytokine activation patterns.

Remarkably, we observed a unique upregulation of PD-1 in about one third of IgA^+^ (and the few IgM^+^) BMPC in response to BCR stimulation, which is characteristic for peripheral B cells after stimulation to counterregulate immune activation (**Figure 5A**) (24), whereas IgA^−^ BMPC showed no substantial increase of PD-1 expression. The upregulation of PD-1 could lead to a regulatory effect on other cells of the immune system as IgA-expressing PC in the murine gut were able to induce regulatory T cells (Treg) in the presence of TGF-β, which suggests that IgA^+^ PC might play an immune regulatory role (31). In the BM of mice, contact of BMPC and Tregs by CD28 and CTLA4 was detected by intravital imaging (32) and signaling *via* the PD-L1/PD-1 axis in Tregs is thought to play a key role in the maintenance and function of the negative regulation of immune responses (33, 34). Our results raise the question if IgA^+^ BMPC are involved in this counterregulation mediated by Tregs. Recently, two different mouse studies described regulatory functions for PC: recirculating IL-10–producing IgA^+^ PC induced in the gut suppress neuroinflammation in a mouse model of autoimmune encephalomyelitis (EAE) (35) and IL-10–producing IgM^+^ PC are natural regulatory PC that can be identified by their LAG-3 expression (36).

Of note, upregulation of PD-1 could be almost completely inhibited by SYK inhibitor entospletinib in BMPC subsets with Ig surface expression (**Figure 5E**). SYK is a prominent kinase expressed in B cells that couples BCR activation to all relevant downstream pathways (37), but also plays a role in CpG-induced activation (38). This finding emphasizes the role of SYK in signal transduction for at least a subset of BMPC and may open innovative possibilities to target these specific cells.

We here identify that IgA-expressing human BMPC are able to respond to antigen encounter with a BCR response translating intracellular signaling and a characteristic PD-1 upregulation independent of CD19 expression. In addition to the emerging phenotypic heterogeneity, the data indicate a functional heterogeneity of mature PC beyond antibody production.

## Data Availability Statement

All data relevant is contained within the article. The sequence data presented in the study has been deposited at the European Genome-phenome Archive (EGA), which is hosted by the EBI and the CRG, under accession number EGAS00001004948.

## Ethics Statement

The studies involving human participants were reviewed and approved by the local ethics committee of Charité Universitätsmedizin Berlin. The patients/participants provided their written informed consent to participate in this study.

## Author Contributions

The concept of the study was planned by AW, AL, and TD. Experiments were conducted, analyzed, and interpreted by AW, ML, IW, and AL. Sample preparation for mRNA Seq, sequencing, and subsequent analysis were done by AJ, AS, GG, and JW. Samples were provided by the Department of CP. AW drafted the manuscript. AL, HEM, AR, and TD edited the manuscript and provided advice. All authors contributed to the article and approved the submitted version.

## Funding

This work was supported by grants from the German Research Foundation Do 491/8-1/2 (SPP Immunobone), TRR130/TP24, Do491/10-1, 11-1 and LI3540/1-1. AS was supported by the German Federal Ministry of Research and Education grant for de.NBI (031L0101D).

## Conflict of Interest

The authors declare that the research was conducted in the absence of any commercial or financial relationships that could be construed as a potential conflict of interest.
