# Supplementary material for: Human IgA-Expressing Bone Marrow Plasma Cells Characteristically Upregulate Programmed Cell Death Protein-1 Upon B Cell Receptor Stimulation
Source: Front Immunol. 2021 Feb 12;11:628923. doi: 10.3389/fimmu.2020.628923 (PMC7907642; doi:10.3389/fimmu.2020.628923)
Supplement: Supplementary file 1 [file DataSheet_1.pdf]

# 1 Supplementary Figures and Tables

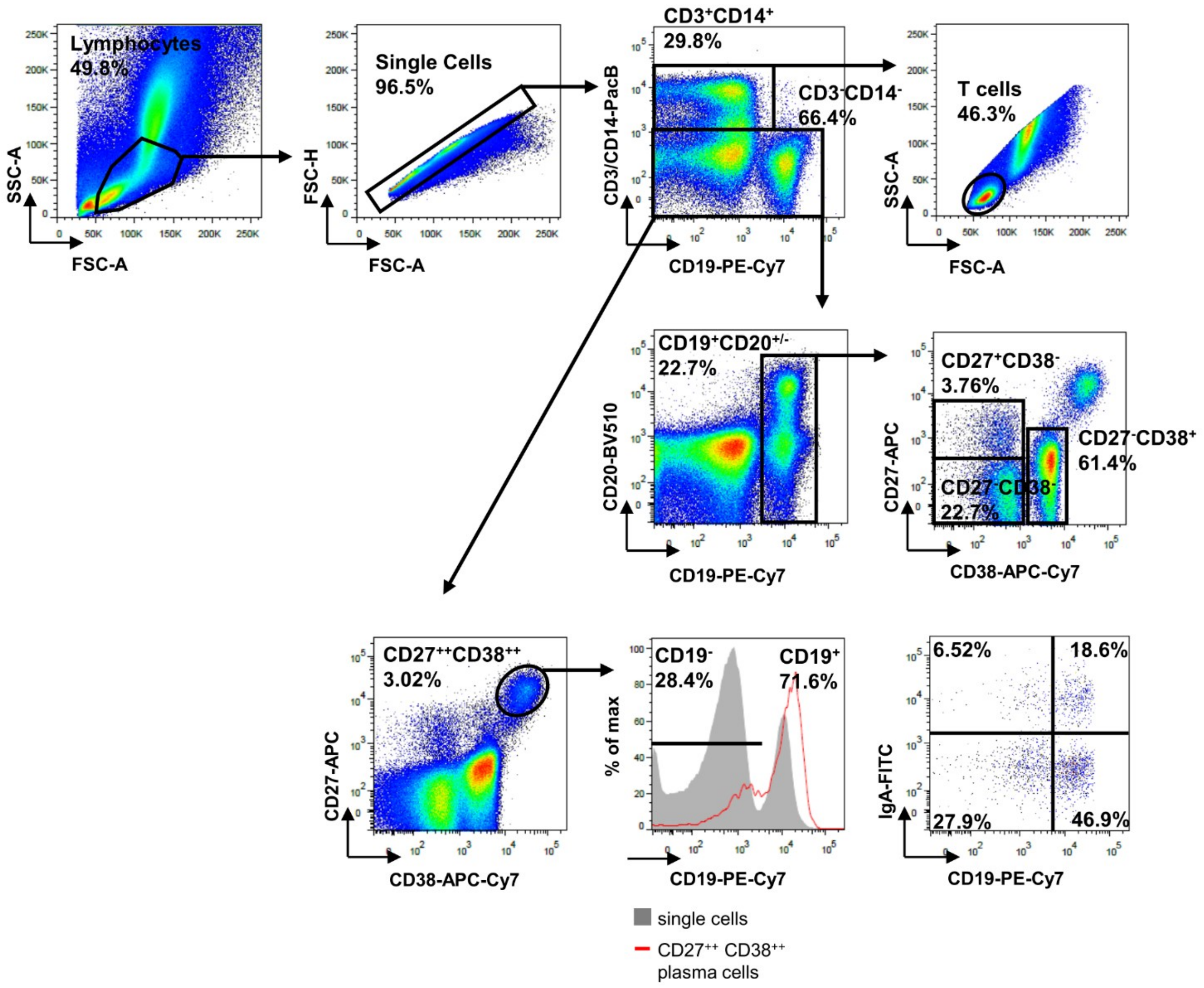


**Figure S1. Gating of BMPC and BMBC subsets.** After gating on lymphocytes and single cells, $CD3^{+}$ and $CD14^{+}$ cells were excluded. In some experiments, T cells were identified by their scatter properties (SSC-$A^{lo}$ and FSC-$A^{lo}$) among $CD3^{+}CD14^{+}$ cells. BMBC were discriminated among $CD3^{-}CD14^{-}$ cells by expression of CD19, in some experiments together with CD20. BC subsets were discriminated by the expression of CD27 and CD38. BMPC were gated by their high expression of CD27 and CD38 out of the $CD3^{-}CD14^{-}$ cells and CD19 expression was assessed in a histogram. Arrows indicate sequential gating.

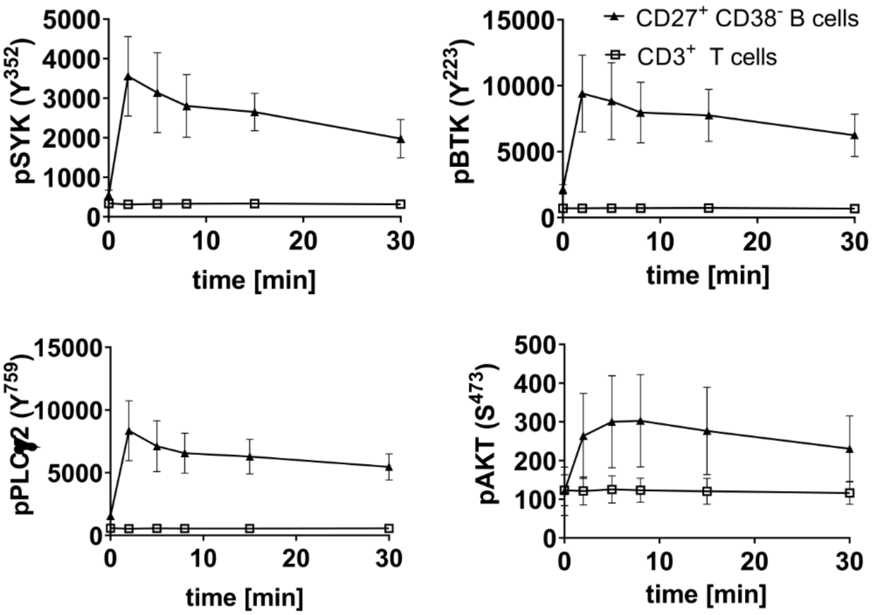


**Figure S2. Stimulation of CD19$^{+}$ and CD19$^{-}$ BM MNCs.** MNCs isolated from the BM were stimulated with anti-BCR F(ab)$_2$ IgM/IgG/IgA at 37°C and stained intracellularly for the respective phosphosites of different PTKs. The MFI of the respective pPTK is shown for CD27$^{+}$CD38$^{-}$ BMBC (positive control) and T cells (negative control), respectively. Data are presented as mean±SD.

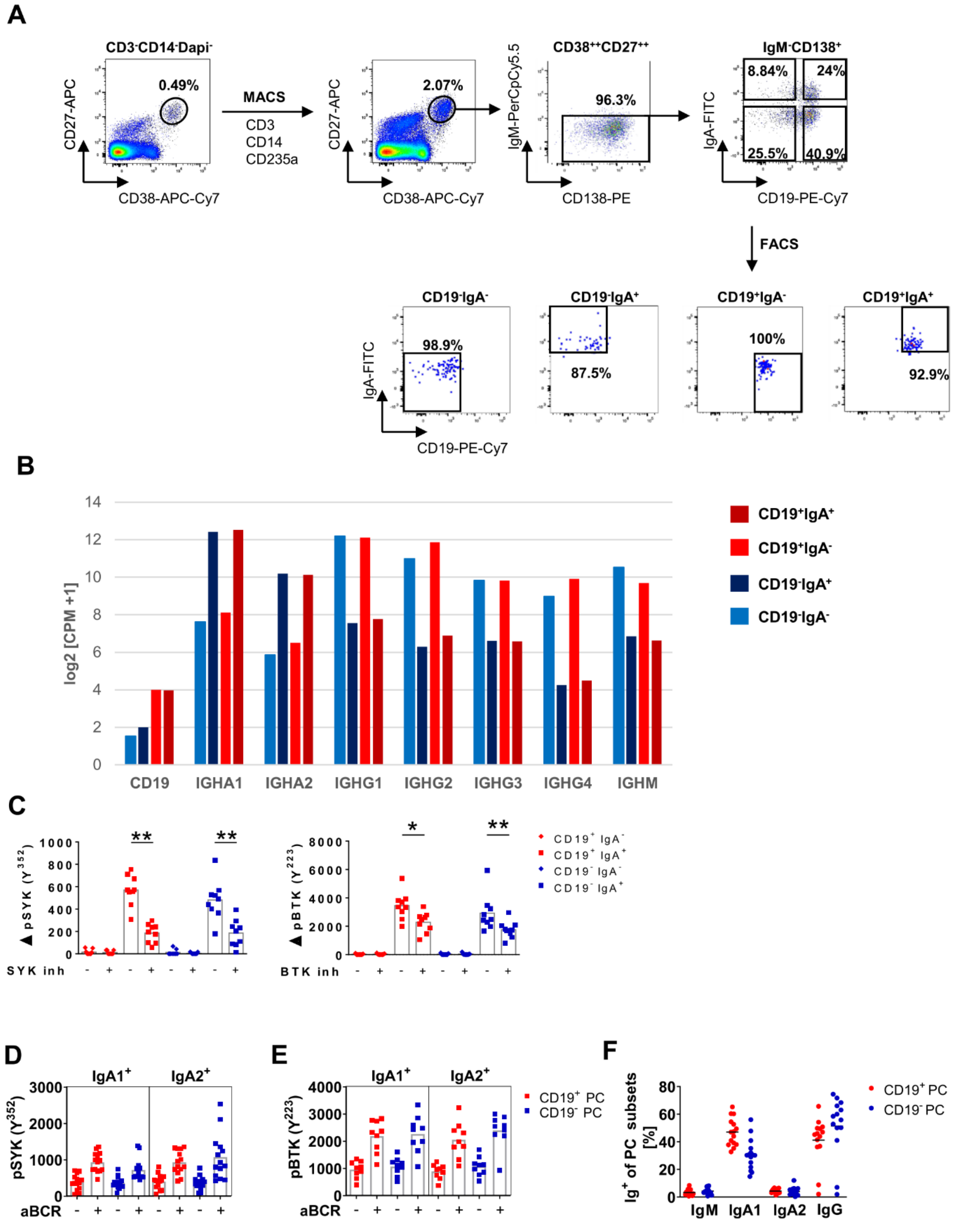


**Figure S3. Ig isotype expression determines BCR responsiveness.** (**A**) Magnetically sorted CD3$^{-}$CD14$^{-}$CD235a$^{-}$ cells were subjected to FACS analysis. BMPC subsets were sorted as CD3$^{-}$CD14$^{-}$Dapi$^{-}$IgM$^{-}$CD27$^{++}$CD38$^{++}$CD19$^{+}$IgA$^{+}$/CD19$^{+}$IgA$^{-}$/CD19$^{-}$IgA$^{+}$/CD19$^{-}$IgA$^{-}$ for mRNA expression analysis

(mRNA Seq); purity of sorted populations was checked by flow cytometry. (**B**) Ig isotype expression on mRNA level in both sorted BM samples. For $CD19^{+}IgA^{-}$, only one sample was analyzed. (**C**) BM MNCs were incubated with SYK inhibitor entospletinib (**left**, n=9) or BTK inhibitor acalabrutinib (**right**, n=9) prior to stimulation with anti-IgA (aIgA) and stained for pSYK $Y^{352}$ or pBTK $Y^{223}$ together with CD19 and IgA. MFI of pSYK $Y^{352}$ and pBTK $Y^{223}$ from a stimulated sample minus unstimulated control (ΔMFI) for the BMPC subsets defined by CD19 and IgA, bar indicates mean, $^{**}p<0.01$, $^{*}p<0.05$ by Friedman-Test with Dunn‘s correction for multiple comparisons. (**D, E**) BM MNCs were stimulated with 30 µg/ml anti-BCR $F(ab)_2$ IgM/IgG/IgA (aBCR) for 5 min and $IgA1^{+}$ and $IgA2^{+}$ cells among $CD19^{+}$ and $CD19^{-}$ BMPC were analyzed for the MFI of pSYK $Y^{352}$ (**D**, n=14) and pBTK $Y^{223}$ (**E,** n=9). Bar indicates mean. Statistical significance was tested with the Friedman Test and Dunn's correction for multiple comparisons, $p>0.05$. (**F**) Frequency of IgM-, IgA1-, IgA2- and IgG-expressing cells among $CD19^{+}$ and $CD19^{-}$ BMPC as determined by intracellular staining. n=15. Statistical significance was tested with the Friedman Test and Dunn's correction for multiple comparisons, $p>0.05$.

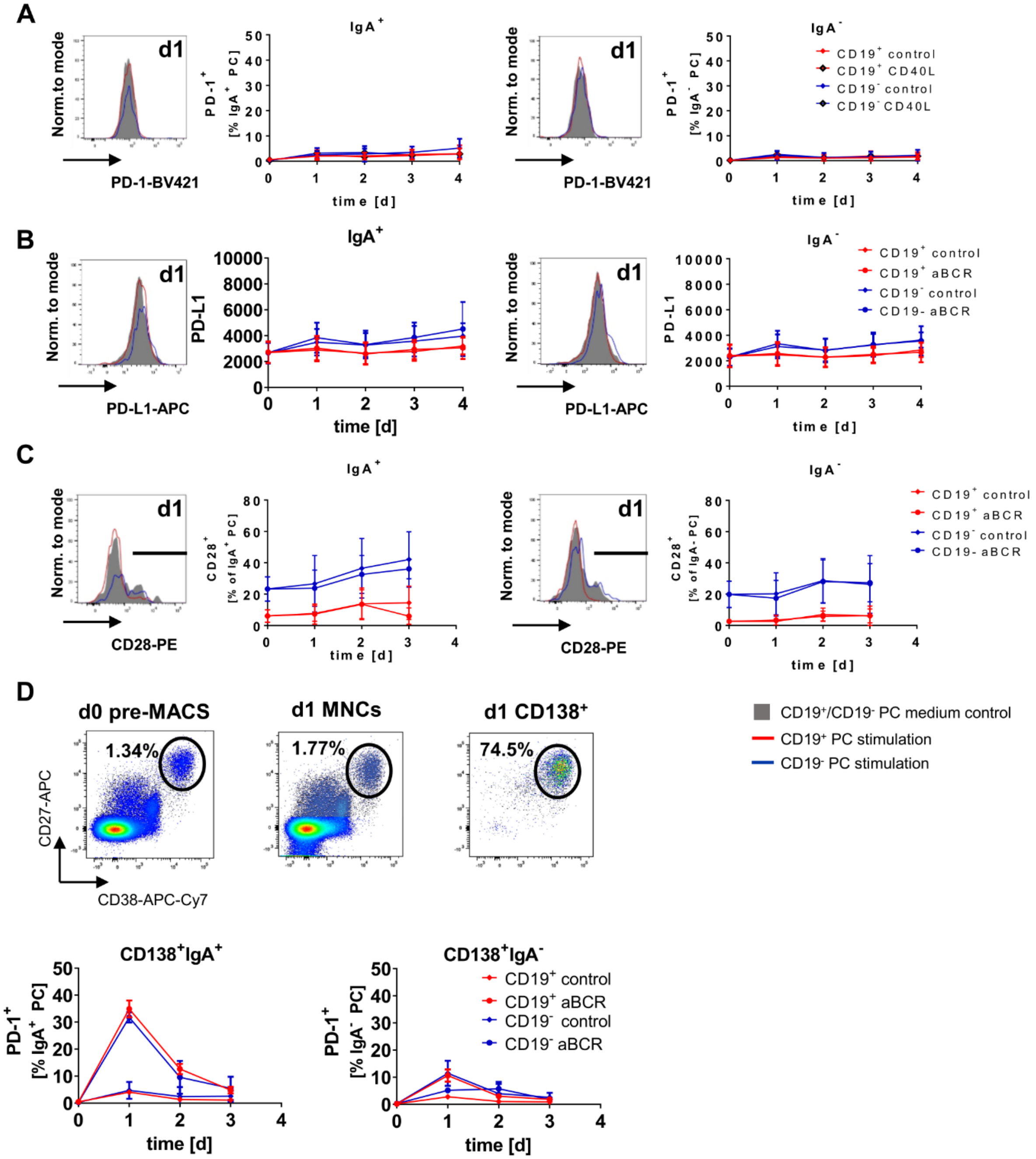


**Figure S4. Stimulation of the BCR does not change the expression of PD-L1, BTLA and CD28 in BMPC.** BM MNCs were cultured for up to four days with either medium (control), 1 µg/ml anti-BCR F(ab)$_2$ IgM/IgG/IgA (aBCR) or 0.5 µg/ml CD40L. Data are presented as mean±SD. (**A**) Representative histogram (d1 control and CD40L) of PD-1 and cumulative PD-1 data of IgA$^+$ (**left**) and IgA$^-$ (**right**) CD19$^{+/-}$ BMPC after CD40L stimulation. n=4-6 for each timepoint. (**B, C**) Representative histogram (d1 control and aBCR) of PD-L1 (MFI, **B**) and CD28$^+$ cells (frequency, **C**) and cumulative data of

$IgA^+$ and $IgA^-$ $CD19^{+/-}$ BMPC after BCR stimulation. n=3-16 for each timepoint. (**D**) Dotplots showing CD27 and CD38 expression of living $CD3^-CD14^-$ cells in MNCs on d0, in MNCs on d1 after incubation with medium and in CD138-enriched BMPC at d1 after incubation with medium (top). CD138-enriched BMPC were cultured for up to three days with medium alone or 1 µg/ml aBCR IgM/IgG/IgA (bottom).

**Table S1. Antibodies used for flow-cytometry and sorting.** PacB: Pacific Blue, BUV: Brilliant UV, PE: Phycoerythrin, PE-Cy7: PE-Cyanine 7, APC: Allophycocyanine, APC-Cy7: APC-Cyanine 7, FITC: Fluorescein isothiocyanate, BV: Brilliant Violet, PerCPCy5.5: Peridinin-chlorophyll protein cyanine 5.5, AF: Alexa Fluor.

| target of antibody | clone | fluorochrome | manufacturer | RRID |
|---|---|---|---|---|
| CD3 | UCHT1 | PacB, BUV395 | BD Biosciences, Heidelberg, Germany | AB_397038, AB_2744387 |
| CD14 | M5E2 | PacB, BUV395 | BD Biosciences, Heidelberg, Germany | AB_397041, AB_2740025 |
| CD19 | SJ25C1 | PE-Cy7, APC-H7, BV711 | BD Biosciences, Heidelberg, Germany | AB_396893, AB_1645728, AB_2737970 |
| | HIB19 | PE-Cy7 | ThermoFisher Scientific, Waltham, USA | AB_1582278 |
| CD20 | HI47 | PacO | Invitrogen ThermoFisher Scientific, Waltham, USA | AB_1500441 |
| | H1FB1 | PerCPCy5.5 | BD Biosciences, Heidelberg, Germany | AB_396990 |
| | 2H7 | BV510 | Biolegend, San Diego, USA | AB_2561941 |
| CD27 | L128 | APC, BV786 | BD Biosciences, Heidelberg, Germany | AB_647368, AB_2744353 |
| CD38 | HIT2 | APC-Cy7, APC, PE | BD Biosciences, Heidelberg, Germany | AB_2561605, AB_398599, AB_395853 |
| CD138 | B-B4 | PE | Miltenyi Biotech, Bergisch Gladbach, Germany | AB_244212 |
| | MI15 | BUV737 | BD Biosciences, Heidelberg, Germany | AB_2738786 |
| SYK | 2D10 | FITC | BD Biosciences, Heidelberg, Germany | AB_394399 |
| | 4D10.2 | PE | Biolegend, San Diego, USA | AB_2565304 |
| pSYK $Y^{352}$ | 17A/P-ZAP70 | PE, AF647 | BD Biosciences, Heidelberg, Germany | AB_396919, AB_396884 |
| PLC-γ2 | K86-1161 | PE | BD Biosciences, Heidelberg, Germany | AB_1645527 |
| PLC-γ2 $Y^{759}$ | K86-689.37 | PE, AF647 | BD Biosciences, Heidelberg, Germany | AB_647226, AB_647139 |
| BTK | 53/Btk | PE | BD Biosciences, Heidelberg, Germany | AB_647183 |

| | | | | |
|---|---|---|---|---|
| pBTK $Y^{223}$ | N35-86 | AF488, PE | BD Biosciences, Heidelberg, Germany | AB_2738981, AB_2721028 |
| panAKT | REA676 | PE | Miltenyi Biotech, Bergisch Gladbach, Germany | AB_2651206 |
| pAKT $S^{473}$ | M89-61 | PE | BD Biosciences, Heidelberg, Germany | AB_1645328 |
| | D9E | APC | Cell Signaling, Cambridge, UK | AB_2797780 |
| IgA | M24A | FITC | Chemicon ThermoFisher Scientific, Waltham, USA | AB_92852 |
| IgA1 | B3506B4 | FITC | Southern Biotech, Birmingham, USA | AB_2796652 |
| IgA2 | IS11-21E11 | PE | Miltenyi Biotech, Bergisch Gladbach, Germany | AB_1036168 |
| IgG | G18-145 | FITC, PE-Cy7 | BD Biosciences, Heidelberg, Germany | AB_396121, AB_10611712 |
| IgM | G20-127 | PerCPCy5.5, BV421 | BD Biosciences, Heidelberg, Germany | AB_10611998, AB_2737681 |
| PD-1 | EH12.1 | BV421 | BD Biosciences, Heidelberg, Germany | AB_11153482 |
| PD-L1 | 29E.2A3 | APC | Biolegend, San Diego, USA | AB_940360 |
| BTLA | MIH26 | PE | Biolegend, San Diego, USA | AB_2043945 |
| LAG-3 | 3DS223H | APC | ThermoFisher Scientific, Waltham, USA | AB_2573186 |
| TIM-3 | F38-2E2 | PE | Biolegend, San Diego, USA | AB_2116576 |
